# Amelioration of popolysaccharide-induced sepsis in rats by free and esterified carnitine

**DOI:** 10.1155/S0962935193000766

**Published:** 1993

**Authors:** Linda L. Gallo, Yan Tian, Zaven Orfalian, Gary Fiskum

**Affiliations:** 1 Department of Biochemistry and Molecular Biology The George Washington University Medical Center 2300 Eye Street N.W. Washington, DC 20037 USA; 2 Sigma Tau SpA Via Pontina Km 30 Pomezia Rome 400-00040 Italy; 3 Department of Emergency Medicine The George Washington University Medical Center 2300 Eye Street N.W. Washington, DC 20037 USA

## Abstract

The purpose of this study was to determine if free or esterified carnitine could alter fatty acid metabolism and ameliorate sepsis in lipopolysaccharide (LPS)-treated rats. Throughout a 96 h observation post-LPS, i.p. administration of both markedly reduced illness and accelerated recovery. Carnitine prevented the acute LPS-induced rise in serum triglycerides (45 ± 6, 59 ± 5 vs. 83 ± 8 mg/ml, p < 0.001), respectively. This difference was accompanied by a significant increase in liver lipogenesis in LPS controls compared to both carnitines and normal rats (6.1 ± 0.3 vs. 3.9 ± 0.5, 4.3 ± 0.5, and 1.8 ± 0.4 μmol/h, respectively, p < 0.04). Compared to normal rats, total liver carnitine was significantly elevated in LPS controls and even higher in the carnitine groups (357 ± 40 vs. 736 ± 38, 796 ± 79, and 1081 ± 21 nmol/g). The data suggest that carnitines may be of therapeutic value in sepsis treatment and one action may be to partition fatty acids from esterification to oxidation.


Mediators of Inflammation 2, S51–S56 (1993)

### Amelioration of lipopolysaccharide-induced sepsis in rats by free and esterified carnitine

Linda L. Gallo,[1,CA] Yan Tian,[1] Zaven Orfalian,[2] and Gary Fiskum[1,3]

[1]Departments of Biochemistry and Molecular Biology and [3]Emergency Medicine, The George Washington University Medical Center, 2300 Eye Street N.W., Washington, DC 20037, USA; [2]Sigma Tau SpA, Via Pontina Km 30,400–00040 Pomezia, Rome, Italy

[CA] Corresponding Author

**THE purpose of this study was to determine if free or esterified carnitine could alter fatty acid metabolism and ameliorate sepsis in lipopolysaccharide (LPS)-treated rats. Throughout a 96 h observation post-LPS, i.p. administration of both markedly reduced illness and accelerated recovery. Carnitine prevented the acute LPS-induced rise in serum triglycerides (45 ± 6, 59 ± 5 *vs.* 83 ± 8 mg/ml, $p < 0.001$), respectively. This difference was accompanied by a significant increase in liver lipogenesis in LPS controls compared to both carnitines and normal rats (6.1 ± 0.3 *vs.* 3.9 ± 0.5, 4.3 ± 0.5, and 1.8 ± 0.4 μmol/h, respectively, $p < 0.04$). Compared to normal rats, total liver carnitine was significantly elevated in LPS controls and even higher in the carnitine groups (357 ± 40 *vs.* 736 ± 38, 796 ± 79, and 1081 ± 21 nmol/g). The data suggest that carnitines may be of therapeutic value in sepsis treatment and one action may be to partition fatty acids from esterification to oxidation.**

**Key words:** Carnitine, Endotoxaemia, Lipogenesis, Lipopolysaccharide, Sepsis, Serum triglycerides

### Introduction

Sepsis and endotoxaemia are accompanied by several disturbances in lipid metabolism. Hypertriglyceridaemia is one of these.[1,2] The elevated serum triglycerides are transported in very low density lipoprotein (VLDL) and accumulate because of increased production in the liver[3] and decreased clearance by peripheral tissues.[1]

Systemic infection is accompanied by increased hepatic lipogenesis and decreased hepatic mitochondrial oxidation of lipids despite a normal or increased availability of free fatty acids.[4] It has been observed in rats rendered endotoxaemic by lipopolysaccharide (LPS), that a decreased capacity for long-chain fatty acyl-CoA oxidation is a consequence of reduced carnitine palmitoyltransferase (CPT) activity.[4]

Another consequence of sepsis is a decreased carnitine concentration in muscle[5] and liver.[4] It is well established that free carnitine is necessary for the transfer of long-chain fatty acids into the mitochondrial matrix for β-oxidation by acting as an acceptor for fatty acyl groups transferred from acyl CoA by CPT,[6] the rate limiting reaction.

Because of this important function for carnitine in normal lipid metabolism and since L-carnitine administration has been shown to increase liver carnitine[7,8] and to counter the symptoms of carnitine deficiency in humans,[9,10] the authors hypothesized that carnitine and/or novel carnitine esters may be effective in normalizing lipid metabolism in sepsis and endotoxaemia.

Further, such a metabolic change which increases energy production and decreases energy storage may ameliorate the illness. This hypothesis was tested in LPS-treated rats administered L-carnitine or the γ-hydroxybutyryl ester of isovalerylcarnitine tartarate (VBT carnitine). The general structure of this ester is provided in Fig. 1.

### Materials and methods

*Experimental protocol:* Male Sprague Dawley rats (Hilltop Labs) weighing 250–300 g at the beginning of the study were held in quarantine for 1 week. During this time the animals were offered rat chow and water *ad libitum* and trained to a 12 h (11 a.m.–11 p.m. dark) reverse light cycle. The experimental protocol shown in Fig. 2 for animal use was approved by the Institutional Animal Care and Use Committee. At −16 h, −8 h, and at zero time experimental animals were injected i.p. with L-carnitine (100 mg/kg body weight) or VBT carnitine (to deliver 100 mg carnitine/kg) in $NaHCO_3$, pH 8.0 (0.8 ml). Controls at these same

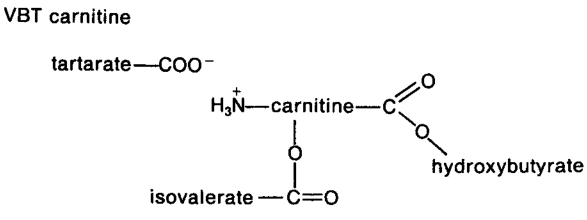

FIG. 1. γ-Hydroxybutyryl ester of isovalerylcarnitine tartarate (VBT carnitine).

© 1993 Rapid Communications of Oxford Ltd

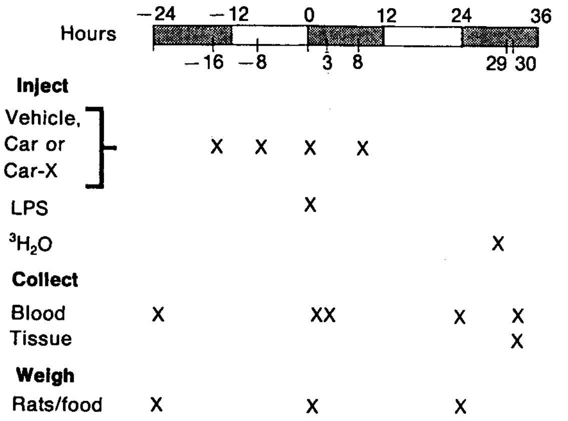

FIG. 2. Experimental protocol. At −16 h, −8 h, and at zero time, animals (17/group) were injected i.p. with $NaHCO_3$ buffer vehicle ± carnitine, VBTcarnitine or the short-chain acids of VBTcarnitine. At zero time all animals except the $NaHCO_3$ controls were injected concurrently with LPS. At 8 h post-LPS the respective groups received a final injection of $NaHCO_3$ buffer ± carnitines or short-chain acids. At −29 h animals (six from each group) were injected i.p. with $^3H_2O$, killed at 30 h, and liver, muscle and blood collected. Tailvein blood was collected at 24 h pre-LPS, at zero time, and at 3 and 24 h post-LPS. Rats and chow were weighed at 24 h pre-LPS and at 24 h intervals. Animal health and survival were monitored regularly for 96 h post-LPS. See methods for specific details. Car = carnitine, Car-X = X-VBTcarnitine, LPS = lipopolysaccharide.

times received the $NaHCO_3$ vehicle ± an amount of tartarate, γ-hydroxybutyrate, and isovalerate equivalent to that in the VBT-carnitine group. At zero time all animals except the $NaHCO_3$ controls were injected concurrently with LPS (24 mg/kg, *E. coli* serotype 0127:B8; Sigma) in $NaHCO_3$ buffer. This amount of LPS had been predetermined to produce 30–50% lethality by 48 h. At 8 h post-LPS the animals received the final i.p. injection of $NaHCO_3$ vehicle ± carnitines as appropriate. At 29 h, fasted rats from each group were selected randomly for measurement of liver lipogenesis, liver mitochondrial oxidative capacity, and tissue carnitine. Each was injected i.p. with $^3H_2O$ (50 mCi, 1Ci/ml, ICN Radiochemicals), a substrate for fatty acid synthesis and 1 h later weighed and killed by decapitation. Liver and skeletal muscle (gastrocnemius) were collected and a small sample of each was immediately frozen in liquid nitrogen and stored at −80°C until analysed and a blood sample was obtained. Mitochondria were prepared from the remaining liver. Tailvein blood from fasting animals was collected at 24 h pre-LPS, at 0 time just prior to LPS injection, and at 3 h and 24 h post-LPS. Rats and chow were weighed at 24 h pre-LPS and at 24 h intervals throughout the study. Animal survival and health were monitored hourly for the first 48 h post-LPS and at regular intervals for an additional 48 h. Health status was assessed by three independent observers and animals were ranked as (a) very sick, if totally inactive, eyes closed, no movement when touched; (b) sick, if some activity and movement when touched but no food consumption; (c) fair, if more active with some food consumption; and (d) normal, if displaying normal activity with much improved food consumption.

*Analytical procedures:*

*Triglycerides.* A commercial kit (GPO-Trinder, Sigma) was used to measure triglyceride levels colorimetrically from blood collected from fasted animals and treated with EDTA. In accordance with the manufacturer's directions, a plasma blank was carried through the procedure with each sample, to correct for haemolysis.

*Lipogenesis* in vivo. Liver samples (1 g) collected 1 h after the i.p. injection of $^3H_2O$ (50 mCi) and 30 h post-LPS were processed precisely as described by Feingold and Grunfeld.[3] Briefly, liver was homogenized and liver lipids were saponified overnight by refluxing in alcoholic KOH. $^{14}$[C]-Oleic acid (20 000 dpm, ICN Radiochemicals) was added as an internal standard to monitor the efficiency of [$^3$H] fatty acid recovery. The nonsaponifiable lipids were extracted by several treatments with petroleum ether (100 ml total). The fatty acid salts in the saponifiable fraction were acidified with concentrated HCl (pH < 2) and the fatty acids extracted into petroleum ether (75 ml) which was evaporated under nitrogen. Fatty acids were resuspended in chloroform (5.0 ml) and an aliquot was added to Scintiverse (Fisher Scientific), a biodegradable liquid scintillant, and radioactivity monitored by liquid scintillation counting. Counts were automatically corrected for [$^3$H] (<0.1%) and [$^{14}$C] (<12.0%) spillover. Calculations were corrected for background counts and recovery (~85%) of the internal standard. The specific activity of the $^3H_2O$, predetermined to be completely equilibriated prior to 1 h, was determined by counting the dpm in 1 ml of plasma from each animal and dividing by mmol of $H_2O$ (52 mmol/ml) per ml of plasma.

*Tissue carnitine.* Free and total carnitine in liver and muscle were determined by the radioisotopic assay described by McGarry and Foster.[11] An aliquot of a protein-free supernatant prepared by perchloric acid treatment of the tissue homogenate was neutralized and assayed for free carnitine. Another aliquot was treated with alkali to hydrolyse short-chain carnitine esters then neutralized and assayed for total acid-soluble carnitine. In both cases, the carnitine containing supernatant was incubated with [$^{14}$C] acetyl CoA (53 mCi/mmole, ICN Radiochemicals) in the presence of carnitine acetyltransferase (Sigma) to yield acetylcarnitine and coenzyme A which was trapped by *N*-ethylmaleimide (Sigma). Unreacted [$^{14}$C] acetyl CoA was removed with the ion exchange resin, Dowex 2 (Sigma) and radioactivity remaining in the supernatant representing tissue carnitine, was determined by liquid scintillation counting. Duplicate portions of each tissue were carried through the procedure and carnitine was assayed in triplicate on each supernatant. Radioactive counts were corrected for background and authentic L-carnitine (Sigma Tau SpA, Italy) served as the standard.

*Mitochondrial respiration.* Mitochondria were prepared from the livers of fasted rats 30 h post-LPS following the procedure of Murphy *et al.*[12] Mitochondrial protein was determined by the method of Lowry *et al.*[13] with bovine serum albumin as standard. The mitochondrial yield was 128 ± 15 mg protein/liver and did not vary significantly among animal groups. State 3 (ADP-stimulated) respiration was measured polarographically with a Clark oxygen electrode (Yellow Springs Instrument Co.) at 37°C essentially according to the methods of Murphy *et al.*[12] Malate (5 mM) and ADP (0.5 mM) were present in all measurements. Glutamate, palmitoylcarnitine, palmitoyl CoA and carnitine were present at concentrations of 5 mM, 0.04 mM, 0.04 mM, and 2 mM, respectively. All chemicals in the assay were products of Sigma.

*Statistical analyses:* Data are expressed as the mean ± S.E.M. Statistical analyses were performed using Student's *t*-test and ANOVA followed by post-ANOVA (Tukey).

### Results

*Animal health and survival:* There was a striking difference in the health of the LPS-treated rats in the presence and absence of carnitine and VBTcarnitine (Table 1). Within 2 h of LPS treatment, five of 17 animals in the LPS group were very sick and eight were sick. In the LPS + carnitine groups all 17 (VBTCar) and 14 (Car) of the animals were in fair to normal health. At 4 h, when all LPS animals were very sick (17), only seven to eight animals in the carnitine groups were very sick. By 30 h the nine survivors from the LPS group ranged from very sick (one), to sick (five), to fair (two), to normal (one). The surviving carnitine animals (nine) and the VBTcarnitine animals (twelve) were restored to fair or normal health. Over the 96 h observation period there were eight deaths in the LPS group, seven deaths in carnitine group and five deaths in the VBTcarnitine group.

*Food consumption and body weight:* Food consumption (Table 2) in the 0–24 h after LPS treatment dropped to <1.0 g/rat with only one rat eating in the LPS group while seven to eight of the animals in both carnitine groups continued to eat. In the 24–48 h after LPS treatment the LPS animals had an average food intake of 8.4 g or 25% of the normal food consumption (34.4 ± 1.1 g) while the carnitine groups consumed an average of nearly twice that amount.

**Table 1.** The variation of survival and health with time after LPS

| Treatment | Time after LPS | | | | | | | | | | | | | | | | | | | | |
|---|---|---|---|---|---|---|---|---|---|---|---|---|---|---|---|---|---|---|---|---|---|
| | 2 h | | | | | 4 h | | | | | 24 h | | | | | 30 h | | | | | 96 h |
| | Dead | VS | S | F | N | Dead | VS | S | F | N | Dead | VS | S | F | N | Dead | VS | S | F | N | Dead |
| LPS | 0 | 5 | 8 | 4 | 0 | 0 | 16 | 1 | 0 | 0 | 7 | 3 | 6 | 1 | 0 | 7 | 2 | 6 | 2 | 0 | 8 |
| LPS + Car | 0 | 0 | 0 | 7 | 10 | 0 | 7 | 10 | 0 | 0 | 3 | 6 | 5 | 3 | 0 | 6 | 0 | 1 | 7 | 2 | 7 |
| LPS + VBTCar | 0 | 0 | 3 | 6 | 8 | 0 | 8 | 8 | 1 | 0 | 5 | 0 | 8 | 4 | 0 | 5 | 0 | 0 | 10 | 2 | 5 |

$n = 17$ rats/group. Scale: VS = very sick; S = sick; F = fair; N = normal. After LPS treatment, animals were monitored hourly for 96 h.

**Table 2.** The variation of food consumption with time

| Treatment | Food consumption (g) | | S | Food consumption (g) | S |
|---|---|---|---|---|---|
| | Day 1, pre-LPS | Day 1, post-LPS | | Day 2, post-LPS | |
| LPS | 27.1 ± 1.6 | 0.4 ± 0.3 | 1/10 | 8.4 ± 2.6 | 4/4 |
| LPS+Car | 28.9 ± 0.9 | 1.6 ± 0.9 | 7/14 | 14.8 ± 2.1 | 4/4 |
| LPS+VBTCar | 30.3 ± 1.4 | 1.5 ± 0.5 | 8/12 | 14.9 ± 3.3 | 6/6 |

Values = Mean ± S.E.M. $n = 17$.
Controls (eight) consumed an average of 27.1 ± 1.1, 35.4 ± 1.1, and 34.4 ± 1.1 g of diet at these respective times.
S = number of survivors consuming food.

**Table 3.** The variation of body weight with time

| Treatment | body weight (g) | | |
|---|---|---|---|
| | Initial | Day 1, post-LPS | Day 2, post-LPS |
| LPS | 277 ± 5 | 257 ± 6 | 256 ± 10 |
| LPS+Car | 279 ± 2 | 262 ± 3 | 261 ± 7 |
| LPS+VBTCar | 277 ± 2 | 271 ± 4 | 271 ± 5 |

Values = mean ± S.E.M. $n = 17$.
Controls (eight) weighed an average of 276 ± 4, 290 ± 4, 287 ± 1 g at these respective times.

Body weights (Table 3) were not significantly different among the LPS, carnitine, and VBTcarnitine groups, although the carnitine and VBTcarnitine groups averaged 5 and 15 g heavier at 24 h (279 ± 2 and 262 ± 3 g) and 48 h (261 ± 7 and 271 ± 5 g) after LPS, respectively. Normal controls at these same times weighed 290 ± 4 g and 287 ± 1 g.

*Serum triglycerides:* Serum triglyceride levels (Table 4) measured at 3 h after LPS treatment had increased an average of 50 mg/dl in the LPS group but only 16 mg/dl in the carnitine group and 27 mg/dl in the VBTcarnitine group. The difference between the LPS and both carnitine groups was statistically significant. By 30 h after LPS treatment serum triglycerides were still elevated by 20 mg/dl in the LPS group but were restored to normal in the carnitine groups.

*Liver lipogenesis* in vivo: Fatty acid biosynthesis *de novo* (Table 5) was significantly higher (> three-fold) in the LPS group than in normal untreated controls. When either carnitine or VBTcarnitine was given to the LPS treated animals, fatty acid biosynthesis significantly decreased to a level which was only 1.4–1.6 fold that in normal controls.

*Tissue carnitine:* The total and free liver carnitine levels (Table 6) in the LPS-treated rats in the presence or absence of the administered carnitines were significantly higher (two- to three-fold) than untreated controls. The total and free carnitine levels were also higher in the LPS animals treated with carnitine (8% and 23%, respectively) or VBTcarnitine (47% and 42%, respectively) than in the LPS groups. The latter difference was statistically significant. In the LPS plus carnitine animals, free carnitine accounts completely for the increase in total carnitine while in the LPS plus VBTcarnitine animals, the increase in total carnitine is due to increases in both free and esterified carnitine (accounts for 66% of the increase).

The total and free muscle carnitine levels (Table 7) in the LPS group were slightly lower, though not significantly, than in the LPS animals treated with carnitine or VBTcarnitine and lower than in normal muscle. Total and free muscle carnitine in the LPS animals treated with carnitine or VBT carnitine were the same as in normal untreated muscle.

*Mitochondrial respiration:* Mitochondrial state 3 respiration was not significantly different among the LPS ± carnitine or VBTcarnitine treatment groups

**Table 4.** The variation of serum triglyceride levels with time

| Treatment | Serum triglyceride level (mg/dl) | | |
|---|---|---|---|
| | Pre-LPS | Post-LPS (h) | |
| | | 3 | 30 |
| LPS | 33 ± 3 | 83 ± 8[a,b] | 53 ± 19 |
| LPS + Car | 29 ± 3 | 45 ± 6[a] | 32 ± 4 |
| LPS + VBTCar | 32 ± 2 | 59 ± 5[a] | 27 ± 3 |

Values = mean ± S.E.M. $n = 17$.
[a]Significantly higher than pre-LPS ($p = 0$).
[b]Significantly higher than LPS + Car and LPS + VBTCar ($p < 0.001$).

**Table 5.** The variation of liver lipogenesis *in vivo*

| Treatment | Lipogenesis | |
|---|---|---|
| | μmol/g/h | μmol/liver/h |
| LPS | 6.1 ± 0.3[a] | 67.8 ± 4.6[a] |
| LPS + Car | 3.9 ± 0.4 | 44.7 ± 6.3 |
| LPS + VBTCar | 4.3 ± 0.5 | 47.6 ± 7.5 |
| None | 1.8 ± 0.4[b] | 24.9 ± 5.5[b] |

29 h post-LPS, rats were injected i.p. with $^3H_2O$ (50 mCi) and 1 h later the liver was removed into liquid nitrogen. Liver lipids were saponified, extracted, and isotope incorporation into fatty acids was measured by liquid scintillation counting.
Values = mean ± S.E.M. for $n = 6$.
[a]Significantly greater than the other experimental groups and untreated control ($p \leq 0.04$).
[b]Significantly less than the experimental groups ($p \leq 0.01$).

**Table 6.** Liver carnitine levels at 30 h post-LPS

| Treatment | Carnitine (nmol/g) | |
|---|---|---|
| | Free | Total |
| LPS | 302 ± 31 | 736 ± 38 |
| LPS + Car | 370 ± 28 | 796 ± 79 |
| LPS + VBTCar | 429 ± 29[b] | 1081 ± 21[b] |
| None | 198 ± 27[a] | 357 ± 40[a] |

Values = mean ± S.E.M. $n = 6$.
Liver was quickly excised into liquid nitrogen from normal controls injected i.p. with vehicle or from experimental animals 30 h after injection with LPS ± carnitines in bicarbonate buffer as described under Methods. Free and total carnitine were determined by radioactivity assay.
[a]Significantly less than all other groups ($p \leq 0.04$).
[b]Significantly greater than all other groups ($p \leq 0.03$).

**Table 7.** Skeletal muscle carnitine levels at 30 h post-LPS

| Treatment | Carnitine (nmol/g) | |
|---|---|---|
| | Free | Total |
| LPS | 679 ± 43 | 829 ± 56 |
| LPS + Car | 729 ± 59 | 921 ± 77 |
| LPS + VBTCar | 807 ± 51 | 963 ± 66 |
| None | 783 ± 81 | 917 ± 81 |

Values = mean ± S.E.M. $n = 6$.
Skeletal muscle was quickly excised into liquid nitrogen from normal controls injected i.p. with vehicle or from experimental animals 30 h after injection with LPS ± carnitines in bicarbonate buffer as described under Methods. Free and total carnitine were determined by assay of radioactivity.

or controls with either glutamate, palmitoylcarnitine or palmitoyl CoA plus carnitine as substrates. Oxygen consumption measured as ng atoms $O_2$/min/mg protein with glutamate in the LPS group was 202.8 ± 8.1 compared to 202.7 ± 15.4, 192.8 ± 8.7, and 179.5 ± 3.0 in the LPS-carnitine, LPS, VBTcarnitine, and controls animals, respectively, with palmitoylcarnitine 138.7 ± 7.1 com pared to 159.6 ± 9.3, 131.5 ± 8.7, and 129.3 ± 6.8, and with palmitoyl CoA plus carnitine 70.8 ± 4.3 compared to 62.9 ± 7.1, 67.4 ± 5.5 and 63.5 ± 1.6, for $n = 5$ animals per group.

### Discussion

Sepsis and endotoxaemia lead to disturbances in lipid metabolism. Notably, hepatic energy production from fatty acid oxidation is suppressed under circumstances where there is an overall body increase in oxygen consumption and an increased tissue demand for oxidative substrates. In this study, the potential of carnitine and a novel carnitine derivative, the γ-hydroxybutyryl ester of isovalerylcarnitine tartarate (VBTcarnitine) to normalize hepatic lipid metabolism was studied in LPS-treated rats.

LPS treatment produced the expected rapid onset of illness described by others (for example see Reference 4). Carnitine and VBTcarnitine markedly delayed and decreased the severity of illness (Table 1). There were no detectable differences in effectiveness between the two. The ester form of carnitine has been demonstrated to be more effective in the treatment of some disorders such as heart muscle[14] and brain ischaemia.[15] Carnitine and VBTcarnitine may have decreased the lethality of LPS as there were seven and five deaths in these groups, respectively and eight deaths in the LPS group. Takeyama *et al.*[4] have reported that carnitine (500 mg/kg body weight given i.p. at 16 h and 30 min pre-LPS) increases the survival rate in LPS-treated rats. Consistent with less illness in the LPS groups treated with carnitine or VBTcarnitine were greater food consumption and less loss of body weight.

Serum triglycerides were significantly lower in both carnitine treatment groups relative to the untreated LPS animals (Table 4) and these results are in agreement with those of Takeyama *et al.*[4] who observed lower serum triglycerides post-LPS in carnitine-treated animals. Decreased hepatic synthesis of fatty acids *de novo* accounts in part, if not fully, for the lower serum triglycerides in the LPS animals treated with carnitine or VBTcarnitine (Table 5). With less fatty acid moving toward esterification, less triglyceride is packaged and secreted in liver VLDL.

Since hepatic fatty acid synthesis and fatty acid oxidation are reciprocal events,[16] the authors suspect that hepatic fatty acid oxidation is stimulated in the LPS groups treated with carnitine or VBTcarnitine. This would be consistent with the observations that hepatic CPT activity declines[4] and malonyl CoA levels increase[4,17] in septic animals. Further, hepatic carnitine levels are reported to increase with carnitine administration in septic animals,[4] carnitine is essential for CPT catalysed translocation of fatty acids into the mitochondria for oxidation,[6] and the decrease in hepatic lipogenesis discussed above would be expected to lower malonyl CoA, a potent CPT inhibitor. In the present study the maximum capacity of isolated liver mitochondria for ATP synthesis (state 3 respiration) with lipid and non-lipid substrates was not different in the LPS groups with or without carnitine treatment. This finding is in agreement with that of Takeyama *et al.*[18] and indicates that any possible change in CPT activity that may occur during septicaemia is insufficient to limit the maximum rate of oxidative energy metabolism measured *in vitro*.

Measurement of liver carnitine levels (Table 6) revealed a significant increase not only in the LPS animals treated with carnitine or VBT carnitine but also in the rats treated only with LPS. This finding was unexpected since liver carnitine levels are reported to decrease in sepsis.[4] However, the observed increase may be controlled by glucagon which increases in sepsis.[19] Injection of glucagon is reported to increase liver carnitine in rats[20] and glucagon also stimulates carnitine uptake in isolated hepatocytes.[21] Elevated liver carnitine in the LPS treated rats as well as in the LPS animals treated with the carnitines diminishes the argument that a differential in liver carnitine levels between the two groups is a determinant of fatty acid metabolism, that is, oxidation *vs.* esterification. Clearly, additional studies will be necessary to determine the mechanism of action of carnitine and carnitine derivatives in ameliorating sepsis.

### References

1. Bagby GJ, Corll CB, Martinez RR. Triacylglycerol kinetics in endotoxic rats with suppressed lipoprotein lipase activity. *Am J Physiol* 1987; **253**: E59–E64.
2. Gallin JI, Kaye D, O'Leary WM. Serum lipids in infection. *N Eng J Med* 1969; **281**: 1081–1086.
3. Feingold KR, Grunfeld C. Tumour necrosis factor-alpha stimulates hepatic lipogenesis in the rat *in vivo*. *J Clin Invest* 1987; **80**: 184–190.
4. Takeyama N, Daisuke T, Nobuaki M, Yasuhide K, Takaya T. Altered hepatic fatty acid metabolism in endotoxicosis: effect of L-carnitine on survival. *Am J Physiol* 1989; **256**: E31–E38.
5. Border JR, Burns GP, Rumph C, Schenk Jr. WG. Carnitine levels in severe infection and starvation: a possible key to the prolonged catabolic state. *Surgery St Louis* 1970; **68**: 175–179.
6. Bremer J. Carnitine metabolism and functions. *Physiol Rev* 1983; **63**: 1420–1480.
7. Brass EP, Hoppel CL. Effect of carnitine or mitochondrial oxidation of palmitoyl-carnitine. *Biochem J* 1980; **188**: 451–458.
8. Brass EP, Hoppel CL. Relationship between acid-soluble carnitine and coenzyme A pools *in vivo*. *Biochem J* 1981; **190**: 495–504.
9. Waber LJ, Valle D, Neill C, DiMauro S, Shug A. Carnitine deficiency presenting as familial cardiomyopathy: a treatable defect in carnitine transport. *J Pediatr* 1982; **101**: 700–705.
10. Worthley LIG, Fishlock RC, Snoswell AM. Carnitine deficiency with hyperbilirubinaemia, generalized skeletal muscle weakness and reactive hypoglycaemia in a patient on long term parenteral nutrition: treatment with intravenous carnitine. *J. Parenter Nutr* 1983; **7**: 176–180.
11. McGarry JD, Foster DW. An improved and simplified radioisotopic assay for the determination of free and esterified carnitine. *J Lipid Res* 1976; **17**: 277–281.
12. Murphy AN, Kelleher JK, Fiskum G. Submicromolar $Ca^{2+}$ regulates phosphorylating respiration by normal rat liver and AS-30D hepatoma mitochondria by different mechanisms. *J Biol Chem* 1990; **265**: 10527–10534.
13. Lowry OH, Rosebrough AL, Farr AL, Randall RJ. Protein measurement with the folin phenol reagent. *J Biol Chem* 1951; **193**: 265–275.
14. Packer L, Reznick AZ, Kagan VE, *et al.* L-Propionyl carnitine, dual mechanism of protection in cardiac ischaemia-reperfusion injury. *FASEB J* 1992; **6**: A1369.
15. Rosenthal RE, Williams R, Bogaert YE, Getson PR, Fiskum G. Prevention of postischaemic canine neurological injury through potentiation of brain energy metabolism by acetyl-L-carnitine. *Stroke* 1992; **23**: 1312–1318.
16. Ide T, Ontko JA. Increased secretion of very low density lipoprotein triglyceride following inhibition of long chain fatty acid oxidation in isolated rat liver. *J Biol Chem* 1981; **256**: 10247–10255.
17. Memon RA, Feingold KR, Moser AH, *et al.* Differential effects of interleukin-1 and tumour necrosis factor on ketogenesis. *Am J Physiol* 1992; **263**: E301–E309.
18. Takeyama N, Itoh Y, Kitazawa Y, Tanaka T. Altered hepatic mitochondrial fatty acid oxidation and ketogenesis in endotoxic rats. *Am J Physiol* 1990; **259**: E498–E505.
19. Marchuk JB, Finely RJ, Groves AC, Wolfe LI, Holliday RL, Duff JH. Catabolic hormones and substrate patterns in septic patients. *J Surg Res* 1977; **23**: 177–182.
20. McGarry JD, Robles-Valdes C, Foster DW. Role of carnitine in hepatic ketogenesis. *Proc Natl Acad Sci USA* 1975; **72**: 4385–4388.
21. Christiansen RZ. Regulation of palmitate metabolism by carnitine and glucagon in hepatocytes isolated from fasted and carbohydrate refed rats. *Biochim Biophys Acta* 1977; **488**: 249–262.

ACKNOWLEDGEMENTS. This research was supported by Sigma Tau SpA Italy.
